# Efficient production of ^2^H, ^13^C, ^15^N-enriched industrial enzyme *Rhizopus chinensis* lipase with native disulfide bonds

**DOI:** 10.1186/s12934-016-0522-7

**Published:** 2016-07-13

**Authors:** Meng Zhang, Xiao-Wei Yu, G. V. T. Swapna, Rong Xiao, Haiyan Zheng, Chong Sha, Yan Xu, Gaetano T. Montelione

**Affiliations:** The Key Laboratory of Industrial Biotechnology, Ministry of Education, School of Biotechnology, Jiangnan University, 1800 Lihu Avenue, Wuxi, 214122 Jiangsu China; State Key Laboratory of Food Science and Technology, Jiangnan University, 1800 Lihu Avenue, Wuxi, 214122 Jiangsu China; Center for Advanced Biotechnology and Medicine, Department of Molecular Biology and Biochemistry, Rutgers, The State University of New Jersey, Piscataway, NJ USA; Department of Biochemistry and Molecular Biology, Robert Wood Johnson Medical School, Rutgers, The State University of New Jersey, Piscataway, NJ USA; Northeast Structural Genomics Consortium, Rutgers, The State University of New Jersey, Piscataway, NJ 08854 USA; Biological Mass Spectrometry Facility, Rutgers, The State University of New Jersey, Piscataway, NJ 08854 USA

**Keywords:** *Rhizopus chinensis* lipase, ^2^H, ^13^C, ^15^N-enriched protein production, Native disulfide bonds, Nuclear magnetic resonance spectroscopy

## Abstract

**Background:**

In order to use most modern methods of NMR spectroscopy to study protein structure and dynamics, isotope-enriched protein samples are essential. Especially for larger proteins (>20 kDa), perdeuterated and Ile (δ1), Leu, and Val methyl-protonated protein samples are required for suppressing nuclear relaxation to provide improved spectral quality, allowing key backbone and side chain resonance assignments needed for protein structure and dynamics studies. *Escherichia coli* and *Pichia pastoris* are two of the most popular expression systems for producing isotope-enriched, recombinant protein samples for NMR investigations. The *P. pastoris* system can be used to produce ^13^C, ^15^N-enriched and even ^2^H,^13^C, ^15^N-enriched protein samples, but efficient methods for producing perdeuterated proteins with Ile (δ1), Leu and Val methyl-protonated groups in *P. pastoris* are still unavailable. Glycosylation heterogeneity also provides challenges to NMR studies. *E. coli* expression systems are efficient for overexpressing perdeuterated and Ile (δ1), Leu, Val methyl-protonated protein samples, but are generally not successful for producing secreted eukaryotic proteins with native disulfide bonds.

**Results:**

The 33 kDa protein—*Rhizopus chinensis* lipase (RCL), an important industrial enzyme, was produced using both *P. pastoris* and *E. coli* BL21 *trxB* (DE3) systems. Samples produced from both systems exhibit identical native disulfide bond formation and similar 2D NMR spectra, indicating similar native protein folding. The yield of ^13^C, ^15^N-enriched r27RCL produced using *P. pastoris* was 1.7 times higher that obtained using *E. coli*, while the isotope-labeling efficiency was ~15 % lower. Protein samples produced in *P. pastoris* exhibit O-glycosylation, while the protein samples produced in *E. coli* were not glycosylated. The specific activity of r27RCL from *P. pastoris* was ~1.4 times higher than that produced in *E. coli*.

**Conclusions:**

These data demonstrate efficient production of ^2^H, ^13^C, ^15^N-enriched, Ile (δ1), Leu, Val methyl-protonated eukaryotic protein r27RCL with native disulfides using the *E. coli* BL21 *trxB* (DE3) system. For certain NMR studies, particularly efforts for resonance assignments, structural studies, and dynamic studies, *E. coli* provides a cost-effective system for producing isotope-enriched RCL. It should also be potential for producing other ^2^H, ^13^C, ^15^N-enriched, Ile (δ1), Leu, Val methyl-protonated eukaryotic proteins with native disulfide bonds.

## Background

Nuclear magnetic resonance (NMR) spectroscopy is a powerful technique for biophysical studies of proteins, providing atom-specific information about protein structure, dynamics and protein interactions. In addition to providing a method complementary to X-ray crystallography for three-dimensional (3D) structure determination, NMR has evolved as a tool for characterizing protein–ligand and protein–protein interactions, measuring affinities and specificities of interactions, identifying binding epitopes, and for characterizing structural rearrangements and allosteric changes induced by ligand binding [1, 2]. Furthermore, NMR relaxation experiments can uniquely characterize and quantify internal dynamics of proteins on nano- to millisecond timescales [3–7].

For most modern NMR spectroscopy methods, isotope labeled (e.g., ^15^N, ^13^C, ^19^F and/or ^2^H) protein samples are required. In the last several years, new methodologies and technologies have been implemented for automated resonance assignments and structure determination [8–10], dynamics studies using nuclear relaxation measurements [11–14], and for studying larger proteins and complexes [15–17]. This creates unique challenges for isotope-enrichment with ^2^H, ^13^C, and/or ^15^N, which is required for determining resonance assignments and for structural and dynamic studies, particularly for larger (>20 kDa) proteins.

The majority of protein NMR studies have used proteins produced in recombinant *E. coli* expression systems [18]. However, many key protein samples needed for biomolecular NMR studies require eukaryotic expression hosts for protein sample production. For these reasons, technologies to produce ^2^H, ^13^C, ^15^N-enriched proteins in eukaryotic expression hosts have high potential impact for protein NMR studies [19, 20]. However, using eukaryotic expression systems, such as *Pichia*, baculovirus, or HEK293 present significant challenges, particularly for ^2^H enrichment [21–26]. In addition, glycosylation in these eukaryotic host systems can be highly heterogeneous. While enzymatic methods are available to remove N-linked glycans, these methods often have very low yields [27, 28]. Methods for removal of O-linked glycans are even less efficient and routine. The large variety of O-glycans dictates the use of several enzymes for removing intact O-linked sugars of a single sample. Thus, chemical deglycosylation is more applicable for removing O-glycans. However, harsh alkali treatment will also disrupt peptide bonds and degrade the protein sample [29]. For such proteins, it may be preferable to produce perdeuterated and Ile (δ1), Leu, Val methyl-protonated protein samples in *E. coli* systems, without glycosylation.

In recent years, various fusion tags have been developed for solubility and stability enhancement and protein folding of partner proteins. Maltose binding protein (MBP) [30] is a highly effective fusion tag for protein sample production in *E. coli* because of its efficient translation initiation, remarkable ability to enhance partner solubility, and mild elution condition [31]. However, soluble MBP-fusion proteins are not always properly folded, especially for those with multiple cysteine residues (and presumably forming disulfide bonds) [32, 33]. According to Nozach et al., for most disulfide-rich proteins they tested, fusions without redox properties (e.g., MBP, GST) could not form correctly folded peptides [34]. Intracellular *E. coli* expression systems for producing ^13^C,^15^N-enriched, perdeuterated, and Ile (δ1), Leu, Val methyl-protonated enriched protein samples are well established and generally highly efficient [8]. However, secreted eukaryotic proteins also often require native disulfide bond formation, which can be challenging to accomplish using these intracellular *E. coli* expression hosts. In particular, reduction and oxidative refolding of secreted eukaryotic proteins into native conformations, while sometimes successful, can often fail or be very challenging [35, 36]. Thus, for certain NMR studies, it is valuable to develop methods that can provide perdeuterated samples of secreted proteins with native disulfide bonds but lacking native glycosylation. These samples can then be used for resonance assignments and initial NMR studies, which can subsequently be extended to studies of the corresponding glycosylated proteins if required.

Here, we describe the production of ^2^H, ^13^C, ^15^N-enriched, non-glycosylated *Rhizopus chinensis* lipase with native disulfide bonds using the *E. coli* BL21 *trxB* (DE3) expression system. RCL is a 33 kDa monomeric protein with extensive industrial enzyme applications [37–40]. ^13^C, ^15^N-enriched RCL was produced using both *P. pastoris* and BL21 *trxB* (DE3) expression systems. We have developed an *E. coli* expression vector coding for a fusion construct in which this lipase gene *proRCL* is fused to the C-terminus of a maltose binding protein (MBP) expression and solubility enhancing tag, *MBP3*-*proRCL*. NMR and mass spectrometry data demonstrate heterogeneous glycosylation of RCL samples produced using the *P. pastoris* expression system. However, using *MBP3*-*proRCL* with the *E. coli* BL21 *trxB* (DE3) system, non-glycosylated, ^13^C,^15^N-enriched MBP-proRCL with native disulfide bonds was produced at high yields. Following removal of the MBP expression enhancement tag, NMR, MS, and enzyme activity studies demonstrate a native-like structure for the resulting purified RCL enzyme. The resulting protocol was then used to produce ^2^H, ^13^C, ^15^N-enriched RCL for NMR studies. These data demonstrate production of a ^2^H, ^13^C, ^15^N -enriched eukaryotic glycoprotein RCL, in non-glycosylated form, with native disulfides, native tertiary structure, and high specific activity using the MBP fusion system combined with *E. coli* BL21 *trxB* (DE3) expression hosts. This expression system provides a potential approach for producing isotope-enriched samples of isotope-enriched secreted, glycosylated eukaryotic proteins in glycan-free forms suitable for NMR studies.

## Methods

### Production of unlabeled and ^13^C, ^15^N-enriched r27RCL in *P. pastoris*

**Fig. 1:**
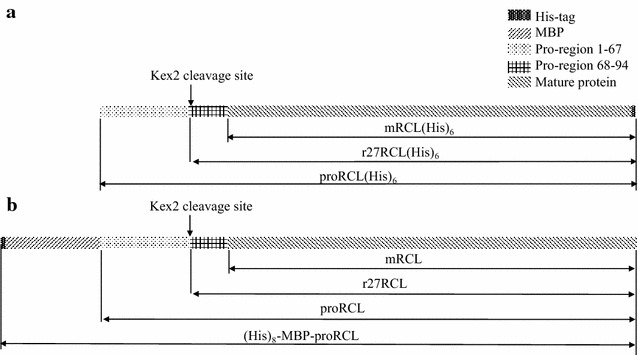
Sketch diagram of various *P. pastoris* r27RCL(His)_6_ (**a**) and *E. coli* r27RCL (**b**) protein constructs

The various forms of RCL produced in *P. pastoris* and *E. coli* are sketched in Fig. 1. Here we adopt the naming conventions of ppRCL and ecRCL for RCL produced in *P. pastoris* and *E. coli*, respectively. The expression and production of ppRCL followed previously described procedures [41]. The PCR-amplified gene products were inserted into pPIC9 K expression vector. The plasmids were electroporated into *P. pastoris* strain GS115, and expressed in BMGY and BMMY media at 30 °C. After 84 h of induction at 28.5 °C, the cell culture was centrifuged at 6000×*g* for 30 min. The supernatant was filtered and purified on an ÄKTA purifier system (GE Co.) using a Ni–NTA column, equilibrated in binding buffer containing 25 mM Tris–HCl pH 8.0, 150 mM NaCl, 20 mM imidazole. The target protein was eluted with 250 mM imidazole. The fractions containing target protein were then dialysed twice against 25 mM Tris–HCl, 150 mM NaCl buffer (pH 8.0), and concentrated using an Amicon Ultra-15 Centricon (Millipore).

The production of ^13^C, ^15^N-enriched ppRCL followed a similar protocol with some modifications [23]. A single colony containing recombinant plasmid was inoculated from a fresh plate to 25 mL of growth medium 1 [100 mM potassium phosphate buffer solution, pH 6.0, YNB (0.34 %, w/v), ammonium sulphate (1 % w/v), biotin (4 × 10^−5^ %, w/v), d-glucose (1 % w/v)], cultivated at 30 °C and 200 rpm for 24 h until the OD_600_ reached 2~6. The cell culture was then centrifuged (3000×*g*, 5 min) and the cells were resuspended in 40 mL of growth Medium 2 [100 mM potassium phosphate buffer solution, pH 6.0, YNB (0.34 %, w/v), ^15^NH_4_Cl (0.02 % w/v), biotin (4 × 10^−5^ %, w/v), ^13^C-d-glucose (0.1 % w/v)]. Cell growth was allowed to continue for 12 h, and stopped by centrifugation (3000×*g*, 5 min). Cells were then washed with a 0.2 % glycerol solution once followed by centrifugation and transferred into 100 mL induction medium [100 mM potassium phosphate buffer solution, pH 6.0, YNB (0.34 %, w/v), ^15^NH_4_Cl (1 % w/v), biotin (4 × 10^−5^ %, w/v)]. Protein expression was induced by the addition of 0.5 % v/v ^13^C-methanol every 12 h. During the induction phase, the pH was adjusted to 6 by 4.4 M KOH and 7.4 M NaOH. After 84 h of induction at 28.5 °C, culture was centrifuged at 6000×*g* for 30 min. The supernatant was the filtered, and purified on an ÄKTA purifier (GE Co.) using a Ni–NTA column as described above. Protein purity was quantified using AlphaView SA, ProteinSimple [42].

### Production of unlabeled and ^13^C, ^15^N-enriched r27RCL in *E. coli*

Expression of MBP-proRCL in *E. coli* BL21 *trxB* (DE3) (Novagen) was started with inoculation a single colony containing recombinant plasmid *MBP3*-*proRCL* from a fresh selection plate to 500 µL LB media at 37 °C and 200 rpm for 4–6 h to obtain a pre-culture. 500 µL of pre-culture was then used to inoculate 50 mL of LB media, and incubated overnight. The full content of this culture was then transferred into a 2 L flask, and incubated at 37 °C and 200 rpm until OD_600_ reached 0.6–0.8 units. The incubation temperature was then decreased to 17 °C, and protein expression was induced by the addition of 1 mM IPTG. After overnight incubation at 17 °C, cells were harvested by centrifugation, the supernatant was discarded, and the pellet was stored at −80 °C. The cell paste was resuspended in 25 mL of binding buffer (50 mM Tris–HCl, 500 mM NaCl, 40 mM imidazole, 1 mM TCEP and 0.02 % NaN_3_, pH 7.5) containing a protease inhibitor cocktail (complete protease inhibitor cocktail, Roche), and sonicated in an ice water bath using a Dual Horn 3/4″ probe (Qsonica, LLC) for 10 min using a 30 s on/30 s off program. The cell debris was cleared by centrifugation at 15,000×*g* for 45 min, followed by filtration (0.45 µm). The supernatant was then loaded onto the ÄKTAxpress system, and a one-step Ni-Gel filtration purification protocol was performed. The 8-His-tagged MBP-proRCL was eluted from the HisTrap HP column (5 mL) using five column volumes of elution buffer (50 mM Tris–HCl, 500 mM NaCl, 500 mM imidazole, 1 mM TCEP and 0.02 % NaN_3_ at pH 7.5). The ÄKTAxpress system collected major peaks into internal storage loop, which was then automatically injected onto a Superdex 200 26/600 gel filtration column equilibrated in low-salt buffer (10 mM Tris–HCl, 100 mM NaCl, 5 mM DTT and 0.02 % NaN_3_ at pH 7.5). The elution was monitored by A_280_ detection, and major peaks were collected in 96-well plate. The elution fractions were analyzed by SDS-PAGE. Fractions containing target protein were pooled together and concentrated using Amicon ultrafiltration concentrators (Millipore). After being concentrated to 8~9 mg/ml, MBP-proRCL was hydrolyzed by Kex2 protease (PeproTech Inc.), using a ratio of 1:100 (protease: protein) at room temperature for 3 h. Purified r27RCL was collected in flow through from Ni–NTA column equilibrated in binding buffer 2 (50 mM Tris–HCl, 500 mM NaCl, 1 mM TCEP and 0.02 % NaN_3_, pH 7.5).

The expression of ^13^C, ^15^N-enriched ecRCL in BL21 *trxB* (DE3) followed a similar, modified protocol. A single colony was picked from a fresh selection plate to 500 µL LB media and incubated for 4–6 h at 37 °C. An aliquot (50 µL) of this rich media preculture was then inoculated to 50 mL of ^15^N- and ^13^C-enriched MJ9 minimal medium [43] and incubated overnight at 37 °C on a shaker (250 rpm). The entire volume of overnight culture was then used to inoculate a 1 L of ^15^N- and ^13^C-enriched MJ9 medium. The culture was incubated at 37 °C (shaking at 250 rpm) until the OD_600_ reaches 0.6–0.8 units and then transferred to 17 °C. Following equilibration at this temperature (~10 min), protein expression was induced with IPTG (1 mM final concentration). After overnight incubation at 17 °C, the cells were harvested by centrifugation and the protein was purified as described above for unlabeled ecRCL purification.

### Production of [^2^H, ^13^C, ^15^N; ^1^H-Ile δ1, Leu-δ,Val-γ]-r27RCL in *E. coli*

^2^H,^13^C,^15^N-enriched ecRCL with ^13^C-enriched, protonated Ile (δ1), Leu, and Val methyl groups ([U -^2^H, ^13^C, ^15^N; ^1^H-Ile δ1, Leu-δ, Val-γ]-r27RCL) was also produced in *E. coli* strain BL21 *trxB* (DE3) using procedures similar to those described in the previous section. Transformed cell cultures were grown at 37 °C in MJ9 minimal media [43] in ^2^H_2_O containing 0.1 % (w/v) (^15^NH_4_)_2_SO_4_ and 0.3 % (w/v) U-^2^H,^13^C-glucose. When the culture reached an OD of 0.35, the culture was moved to 17 °C, and [U-^13^C_4_, 3,3-^2^H_2_]-α-ketobutyrate (50 mg/l), [U-^13^C_5_, 3-^2^H]-α-ketoisovalerate (CIL Inc.) (100 mg/l) added for methyl labeling [44]. Protein expression was induced overnight by addition of IPTG to a final concentration of 1 mM. Cells were harvested by centrifugation at 6000×*g* for 30 min. [^2^H, ^13^C, ^15^N; ^1^H-Ile δ1, Leu-δ, Val-γ]-ecRCL was then purified as described above for ^13^C, ^15^N-labeled ecRCL.

### Lipase activity and protein concentration measurements

Lipase activity was measured according to Kordel et al. [45]. A 7.9 mM solution of *p*NPP in isopropanol and 50 mM PBS buffer pH 8.0, containing 1.16 g/L sodium deoxycholate and 0.56 g/L arabic gum was mixed prior to use with a rate of 1:9 as substrate. The reaction was carried out by adding 0.1 mL of 3–4 μM ppRCL or ecRCL solution into 2.4 mL of the substrate mixture. The absorbance at 410 nm against a blank was monitored using a SpectraMax® Plus 384 (molecular devices) after reacted at 40 °C for 2 min. One enzyme unit was defined as the amount of enzyme releasing 1 μmol of *p*-nitrophenol per minute under the assay conditions (pH 8.0, 40 °C). The protein concentration was determined by Pierce BCA protein assay kit (Thermo Scientific) using bovine serum albumin (BSA) as a standard.

### Disulfide bond mapping and glycosylation analysis

For disulfide bond mapping, 56 µg each of ppRCL and ecRCL were incubated with 10 mM iodoacetamide (ΙΑΜ), 6 M urea in Tris–HCl buffer pH 7.5 at room temperature for 1 h to alkylate cysteines. After alkylation, excess iodoacetamide was removed using 10 kDa cutoff ultrafiltration device. The samples were then hydrolyzed into peptide fragments with chymotrypsin (molarities were chymotrypsin: protein = 1:25) in ammonium acetate buffer pH 6.0. Two equal fractions were created. The first fraction was reduced with 10 mM TCEP to reduce the disulfide bonds and create free cysteine groups. The second fraction was served as a disulfide-intact control. Peptides were reconstituted in 0.1 % TFA and analyzed by LC–MS/MS on a Dionex RSLC system (ThermoFisher, San Jose, CA) interfaced with a LTQ Orbitrap Velos (ThermoFisher, San Jose, CA) online with a Thermo LTQ Orbitrap Velos mass spectrometry (Thermo Fisher Scientific, San Jose, CA). The peptide mixtures were loaded onto self-packed 100 µm × 2 cm trap packed with Magic C18AQ, 5 µm 200 A (Michrom Bioresources Inc, Aubum, CA) and washed with Buffer A (0.2 % formic acid) for 5 min at a flow rate of 10 µl/min. Separation was achieved with an analytical reverse-phase chromatography column (Magic C18AQ, 3 µm 200 A, 75 µm × 50 cm) and peptides were fractionated at 300 nl/min with a multi-stepped gradient [4–15 % buffer B (0.16 % formic acid 80 % acetonitrile) in 10 min and 15–50 % buffer B in 40 min]. Mass spectrometry data was acquired using a data-dependent acquisition procedure with a cyclic series of a full scan acquired in Orbitrap with resolution of 60,000 followed by MS/MS scans (CID 35 % of collision energy) of 20 most intense ions with a repeat count of two and the dynamic exclusion duration of 60 s. The MS/MS data were searched against a custom fasta database including target protein sequences using X!tandem (SLEDGEHAMMER (2013.09.01), thegpm.org) with carbamidomethylation on cysteine as fixed modification and oxidation of methionine and deamidation on asparagine as variable modifications using a 10 ppm precursor ion tolerance and a 0.4 Da fragment ion tolerance.

For the glycosylation analysis, 10 µg of ppRCL was first separated by SDS-PAGE. Gel bands were cut and divided into two fractions: one fraction was in-gel digested by trypsin, whereas the other fraction was digested with Endoproteinase Asp-N. For Asp-N digested sample, a portion was digested again with trypsin for 2 h (Asp-N + trypsin sample), while the other sample’s portion was also digested with chymotrypsin in solution. For this experiment, 10 μg of protein was denatured by addition of 6 M guanadine HCl, reduced with 20 mM DTT at 60 °C for 30 min, alkylated with 40 mM iodoacetamide, and incubated for 45 min at room temperature in the dark. Afterwards, the samples were desalted using spin filter with MWCO10KD, followed by digestion with chymotrypsin on the membrane at room temperature for 18 h and eluted by passing the filter again. All samples were analyzed by nano-LC–MS/MS. Data was manually interpreted.

### NMR sample preparation, data collection and processing

^13^C, ^15^N-enriched pp27RCL and ecRCL samples were prepared at 0.5 mM protein concentrations in 0.1 M sodium phosphate buffer pH 6.0 containing 10 % ^2^H_2_O and 50 µM 4,4-dimethyl-4-silapentane-1-sulfonate sodium salt (DSS). The [^2^H, ^13^C, ^15^N; ^1^H-Ile δ1, Leu-δ, Val-γ]-ecRCL was prepared at 0.12 mM concentration using the same pH 6.0 sodium phosphate buffer. [^1^H, ^15^N]-TROSY-HSQC and [^1^H, ^13^C]-HSQC 2D spectra were collected on Bruker Avance II 800 MHz spectrometer at 35 °C. NMR data were processed and analyzed with NMRPipe, NMRDraw [46] and SPARKY [47]. NMRPipe was used for data processing, and both NMRDraw and SPARKY for spectral display and peak analysis.

## Results

### Unlabeled and isotope-enriched r27RCL expression in *P. pastoris* and *E. coli*

**Fig. 2:**
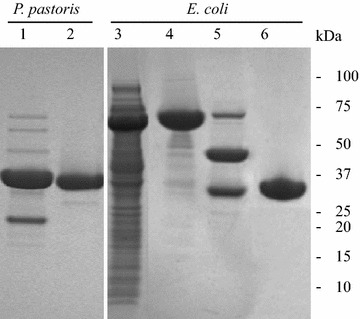
Expression and purification of r27RCL by *P. pastoris* and *E. coli. 1* soluble protein, *2* r27RCL(His)_6_ after HisTrap column, *3* soluble protein, *4* MBP-proRCL after one-step HisTrap-size-exclusion chromatography, *5* after Kex2 cleavage, *6* r27RCL after HisTrap column

To compare the levels of heterologous ppRCL and ecRCL expression, the expression levels and enzymatic activities were compared. As described in detail in the “Methods” section, after 84 h of induction by methanol, proRCL was expressed, hydrolyzed by Kex2 protease (which is expressed naturally by *P. pastoris*) at the recognition site Lys–Arg between −29 and −28 of the prosequence, and secreted into culture supernatant as ppRCL (Fig. 1). The supernatant (Fig. 2, lane 1) was purified using a Ni–NTA column with gradient elution. Purity was over 95 % (Fig. 2, lane 2) as analyzed by AlphaView SA, ProteinSimple [42]. When produced in *P. pastoris*, the heterologous protein secreted into the culture was relatively pure and the yield was 124.8 ± 5.3 mg/L after one-step Ni–NTA purification.

For the production of ecRCL, soluble fusion protein MBP-proRCL (Fig. 2, lane 3) was purified by sequential Ni–NTA and gel filtration chromatography (Fig. 2, lane 4), followed by tag cleavage by Kex2 protease (Fig. 2, lane 5). The N-terminal His_8_-MBP tag together with 67 amino-acid residues of the pro-region of RCL (Fig. 1), and the residual un-hydrolyzed fusion protein, was then removed using a Ni–NTA column. Purification buffers included 1 mM TCEP or 5 mM DTT, which may prevent the formation of dimer or polymer but were not sufficient enough to reduce the disulfide bonds. Pure ecRCL (Fig. 2, lane 6) was pooled together from flow-through fractions. The purity was over 95 % as quantified using AlphaView SA, ProteinSimple [42]. The expression level of MBP-proRCL in *E. coli* was 150.2 ± 3.8 mg/L, but after the sequential Ni–NTA and gel filtration purifications, Kex2 cleavage and subsequent Ni–NTA purification, the final ecRCL yield was 18.7 ± 1.4 mg/L.

Validation of ppRCL and ecRCL were performed by MALDI-TOF mass spectrometry. The ppRCL was partly glycosylated, while the major peak was not glycosylated. Accordingly, the major peak was used for the measurement of molecular weight. The measured molecular weights of ppRCL and ecRCL were 33339.6 ± 25.1 and 32280.8 ± 23.8 Da, in agreement with the expected corresponding theoretical molecular weights of 33325.6 and 32271.5 Da, respectively.

**Fig. 3:**
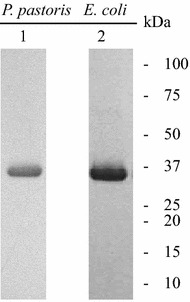
SDS-PAGE of purified ^13^C, ^15^N-enriched r27RCL by *P. pastoris* and *E. coli. 1* ^13^C, ^15^N-enriched ppRCL, *2* ^13^C, ^15^N-enriched ecRCL

We next compared the production of isotope-enriched r27RCL using the *P. pastoris* and *E. coli* expression systems. Both expression levels and the completeness of isotope incorporation were assessed. As described in detail in the Materials and Methods section, after 12 h of cell adaptation in isotope-enriched growth medium, and 84 h of induction using ^13^C-methanol [48], proRCL was expressed, hydrolyzed by Kex2 protease of *P. pastoris* and secreted into culture supernatant. The supernatant was purified following the same protocol used for the unlabeled protein, providing purity over 95 % (AlphaView SA, ProteinSimple [42]). For the production of ^15^N, ^13^C-labeled ecRCL, the fusion protein MBP-proRCL was produced in MJ9 media [43], and purified as described above. The SDS-PAGE gel was similar to the one with unlabeled protein, as shown in Fig. 3, lane 1, 2. For production of ^13^C, ^15^N-enriched ppRCL, the yield was 25.6 ± 3.1 mg/L after one-step Ni–NTA purification. The expression level of ^13^C, ^15^N-enriched MBP-proRCL in *E. coli* was 121.5 ± 4.1 mg/L, but the final ecRCL yield was only 15.3 ± 2.5 mg/L. The percentages of the labeled species were estimated using the difference between the observed mass and the theoretical average mass. The isotope incorporation completeness was 83.6 ± 3.2 % for ppRCL, while it was 99.2 ± 0.2 % for ecRCL (Table 1).

**Table 1:** Production of isotope-enriched r27RCL in *P. pastoris* and *E. coli*

Expression systems	Expressed proteins	Expression level (mg/l)	Enrichment efficiency
*P. pastoris*	^13^C, ^15^N-enriched ppRCL	25.6 ± 3.1	83.6 ± 3.2 %
*E. coli*	^13^C, ^15^N-enriched ecRCL	15.3 ± 2.5	99.2 ± 0.2 %

### Validation of native disulfide bond formation

**Fig. 4:**
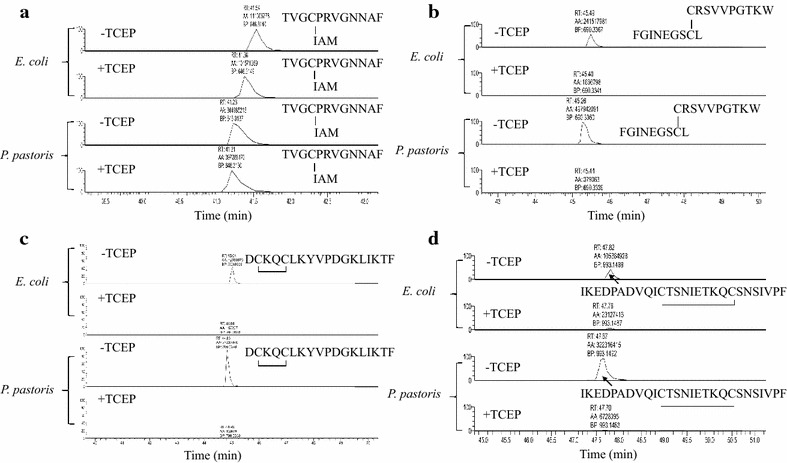
Disulfide bond mapping of r27RCL(His)_6_ from *P. pastoris* and r27RCL from *E. coli.* LC–MS fragments of *P. pastoris* and *E. coli* w/o TCEP treatment **a** C204 [alkylated with iodoacetamide (IAM)]; **b** C56–C295 disulfide bond pair; **c** C67–C70 intra-disulfide bond peptide; **d** intra-disulfide bond peptide indicating C262-C271

Correct formation of native disulfide bonds is critical for stabilizing protein structures and maintaining protein function. One of the major advantages of *P. pastoris* over *E. coli* is that *P. pastoris* is capable of producing secreted proteins with native disulfide bonds [49]. The *E. coli* trxB strain lacks thioredoxin reductase, which allows formation of disulfide bonds within the *E. coli* cytoplasm [50]. To verify if *E. coli* expressed correctly folded ecRCL with native disulfide bonds, enzymatic digestion followed by mass spectrometry (MS/MS) detection was used for disulfide linkage assignment. The formation of one disulfide bond results in a 2-Da reduction of molecular weight, which can be distinguished by high-resolution mass spectrometry. Native disulfide bonds linking peptide fragments ^56^CRSVVPGTKW^65^ and ^288^FGINEDSCL^296^ (Fig. 4b, −TCEP) and forming intra-fragment disulfide bonds in peptides ^66^DCKQCLKYVPDGKLIKTF^83^ (Fig. 4c, −TCEP), and ^252^IKEDPADVQICTSNIETKQCSNSIVPF^278^ (Fig. 4d, −TCEP) were detected in control analyses, which were not treated with the disulfide reducing agent TCEP. The disulfide bonds linking these peptide fragments, and intra-fragment disulfide bonds, were cleaved when treated with TCEP. Thus reduction of disulfide bonds linking peptide fragments resulted in two peptide fragments, and reduction of intra-fragment disulfide bonds resulted in a 2-Da reduction of molecular weight. Since molecular weight shift changes the retention time, those reduced peptides were not detected in the selected molecular weight windows (Fig. 4b, 4c, 4d, +TCEP) with the same retention times as in control groups. As described in the methods section, free cysteines were alkylated during the treatment. Alkylated C204 fragment (^201^TVGCPRVGNNAF^212^) was detected in ppRCL and ecRCL samples (Fig. 4a), indicating that C204 is a free cysteine in both protein samples. These results indicated that native disulfide bonds are formed between cysteines C56–C295, C67–7C0 and C262–C271 in both ppRCL and ecRCL.

### [^1^H, ^15^N]-TROSY-HSQC and [^1^H, ^13^C]-HSQC 2D NMR Spectra

**Fig. 5:**
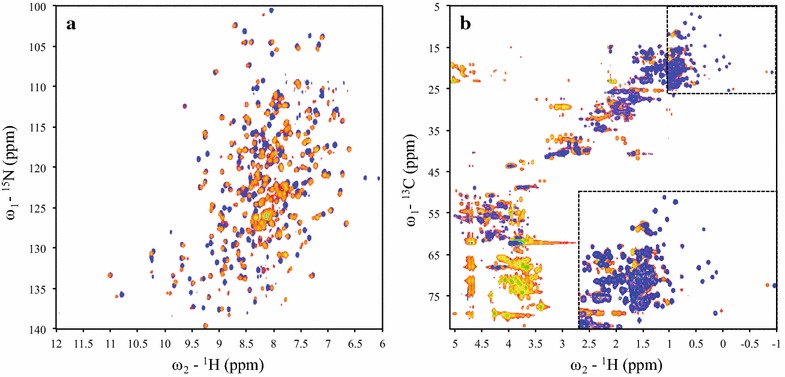
800 MHz NMR spectra of ^13^C, ^15^N-enriched r27RCL(His)_6_ expressed in *P. pastoris* (*red*), r27RCL expressed in *E. coli* (*blue*) at 35 °C and pH 6. **a** [^1^H, ^15^N]-TROSY-HSQC spectra, **b** [^1^H, ^13^C]-HSQC spectra

[^1^H, ^15^N]-TROSY-HSQC NMR spectra of ^13^C, ^15^N-enriched ppRCL and ecRCL showed good resonance dispersion, characteristic of a well-folded, globular proteins (Fig. 5a). Most resonances of proteins produced in either expression system have essentially identical chemical shifts, indicating that the structures of ppRCL and ecRCL are largely identical. However, because of the lower isotope-enrichment levels, the number of backbone ^1^H-^15^N correlation peaks identified in the [^1^H, ^15^N]-TROSY-HSQC spectra of ppRCL was only 213 out of an expected 276 backbone amides. On the other hand, 271 [^1^H, ^15^N]-TROSY-HSQC backbone correlation peaks were identified in the ^13^C,^15^N-enriched ecRCL sample. The ^1^H and ^15^N resonances in spectra of ppRCL are generally broader than those of ecRCL. This may be due to the hexa-His C-terminal tag and/or residual glycosylation (described below) of ppRCL.

As can be observed from the superimposed [^1^H-^13^C]-HSQC NMR spectra of ppRCL and ecRCL (Fig. 5b), ppRCL sample also has many extra ^1^H–^13^C correlation peaks in the region 3.5–4.5 ppm in the ^1^H dimension and 65–80 ppm in the ^13^C dimension. These are assigned to glycans associated with the protein sample, demonstrating that ppRCL protein using the methods outlined in this work remains partially glycosylated.

### Glycosylation analysis

**Fig. 6:**
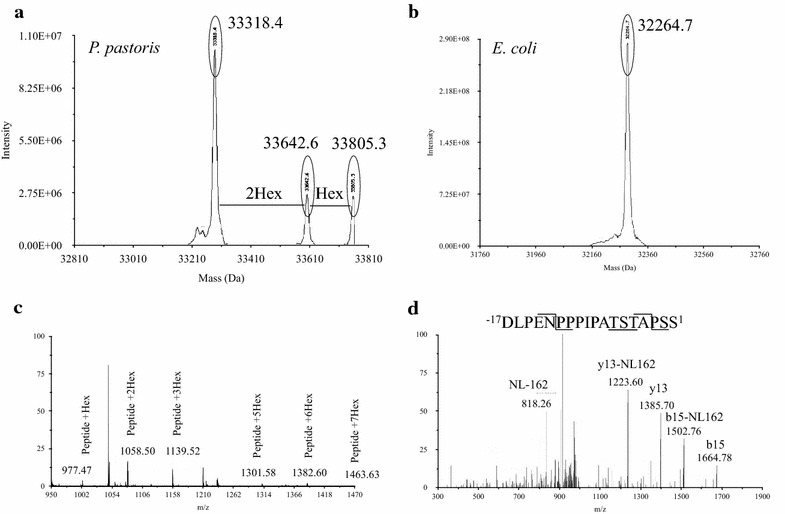
MS spectra of r27RCL from *P. pastoris* and *E. coli* and LC–MS/MS tandem mass spectrum of peptide sequences obtained by enzymatic digestion **a** r27RCL(His)_6_ from *P. pastoris*, **b** r27RCL from *E. coli*, **c** ^−17^DLPENPPPIPATSTAPSS^1^ peptide from AspN digested *P. pastoris* r27RCL(His)_6_, **d** MS/MS spectrum of peptide ^−17^DLPENPPPIPATSTAPSS^1^

Protein glycosylation is a common post-translational modification, which can play key roles in the function of enzymes and regulation of their enzymatic activities. 2D [^1^H, ^13^C]-HSQC spectra of ppRCL clearly indicated some glycosylation (Fig. 5b). To further profile the glycan microheterogeneity, purified ppRCL and ecRCL were analyzed by electrospray mass spectrometry (Fig. 6a, b). The spectrum of ecRCL exhibits only one peak of 32264.7 Da (Fig. 6b). However, ppRCL exhibits multiple MALDI-TOF mass spectrometry peaks, with molecular weights of 33318.4 Da (peak 1), 33642.6 Da (peak 2), and 33805.3 Da (peak 3), respectively (Fig. 6a). These mass increments correspond to masses of hexose units (162 Da). Furthermore, after digestion of ppRCL with Endoproteinase Asp–N and analysis by LC–MS/MS, the glycosylation site was revealed to be a non-hydrolyzed O-glycosylation mapped to a Ser or Thr residue within the polypeptide segment ^−17^DLPENPPPIPATSTAPSS^1^ (Fig. 6c, d).

### Expression level and activity comparisons

As an enzyme, specific activity is an essential characteristic. To further compare the specific activity of r27RCL from *E. coli* and *P. pastoris*, unlabeled proteins were both buffer exchanged to 25 mM Tris–HCl, 150 mM NaCl, pH 8.0 buffer, which is the optimum pH for r27RCL. Lipase activity was measured at 40 °C for 2 min. The specific activities were 283.52 ± 4.28 and 118.48 ± 0.45 U/mg for ppRCL and ecRCL, respectively.

### Expression and NMR studies of [^2^H, ^13^C, ^15^N; ^1^H-Ile δ1, Leu-δ, Val-γ]-r27RCL

**Fig. 7:**
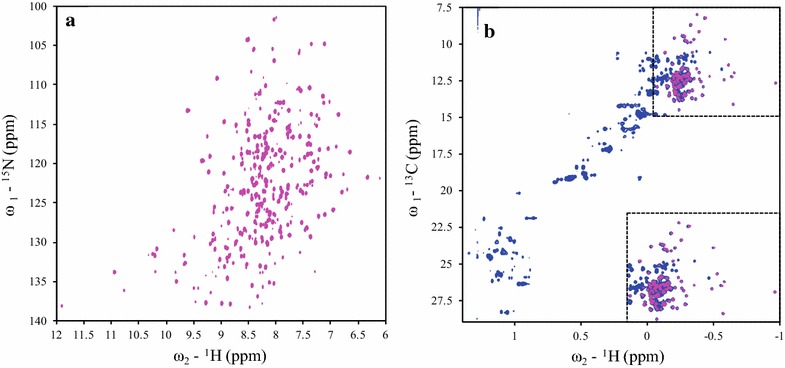
800 MHz NMR spectra of [^2^H, ^13^C, ^15^N; ^1^H-Ile δ1, Leu-δ, Val-γ]-r27RCL (*magenta*) and ^1^H, ^13^C, ^15^N-enriched r27RCL (*blue*) expressed in *E. coli* at 35 °C and pH 6. **a** [^1^H, ^15^N]-TROSY-HSQC spectra, **b** [^1^H, ^13^C]-HSQC spectra

Samples of perdeuterated [^2^H, ^13^C, ^15^N; ^1^H-Ile δ1, Leu-δ, Val-γ] -enriched r27RCL were also prepared in the *E. coli* BL21 *trxB* (DE3) expression system. The growth rate of adapted cells in ^2^H,^13^C,^15^N-enriched MJ9 medium in ^2^H_2_O was relatively slow compared with those in ^13^C, ^15^N-enriched MJ9 medium in H_2_O. It took 8 h before OD_600_ reached 0.35 units, at which point the ^13^C-^1^H methyl labeling precursors were added. The yield of purified fusion protein MBP-proRCL and r27RCL were 51.2 and 4.3 mg/L, respectively. Based on the [^1^H, ^13^C]-HSQC NMR spectrum (Fig. 7b, magenta), it appears that this protein sample is fully perdeuterated except for the targeted methyl groups, as there is no indication of ^1^H–^13^C correlation peaks except those of the Ile δ1 methyl and the Leu-δ, Val-γ isopropyl methyl groups. 96 out of 106 Ile δ1 methyl and Leu-δ/Val-γ isopropyl methyl groups were observed. Compared with the 2D [^1^H, ^13^C]-HSQC spectrum of ^13^C, ^15^N sample (Fig. 7b, blue), the spectrum of [^2^H, ^13^C, ^15^N; ^1^H-Ile δ1, Leu-δ, Val-γ]-r27RCL sample also showed significant improvement of resolution and reductions of peak linewidths in both ^1^H and ^13^C dimensions. Furthermore, comparing the one-dimensional slices of [^1^H, ^15^N]-TROSY-HSQC spectra of ^13^C,^15^N-enriched (Fig. 5a, blue) and ^2^H,^13^C,^15^N-enriched ecRCL (Fig. 7a), the NMR resonances were significantly shaper in perdeuterated sample spectrum. For example, the proton linewidths for the amide ^15^N–^1^H correlation resonance of Gly7 are ~26.4 Hz for the ^13^C,^15^N-enriched sample, and ~20.6 Hz for ^2^H,^13^C,^15^N-enriched ecRCL. Hence, [^2^H, ^13^C, ^15^N; ^1^H-Ile δ1, Leu-δ, Val-γ] -enriched ecRCL with native disulfide bonds can be produced in sufficient quantities for NMR studies using the *E. coli* BL21 *trxB* (DE3) expression system.

## Discussion

Although bacterial host systems have been developed that can sometimes be used successfully to produce protein samples with native disulfide bonds [36, 51, 52], production of isotope-enriched proteins with native disulfide bonds can present a significant challenge which needs to be overcome for NMR studies. In particular, while the *E. coli* BL21 *trxB* (DE3) strain has been used extensively for producing proteins, including unlabeled proteins for NMR studies [53] and ^15^N, ^13^C-enriched proteins using specialized Celtone enriched media for triple-resonance NMR studies [54], our study demonstrates the use of BL21 *trxB* (DE3) for production of ^2^H, ^13^C, ^15^N-enriched RCL with native disulfide bonds in defined MJ9 minimal media. This is the first example of using the *E. coli* BL21 *trxB* (DE3) expression system to produce a disulfide-containing, perduterated [^2^H, ^13^C, ^15^N; ^1^H-Ile δ1, Leu-δ, Val-γ]-enriched eukaryotic protein. The technology in enabled by using the MBP fusion system. This approach is especially valuable for larger proteins (>20 kDa), since perdeuteration is required to suppress nuclear relaxation properties of the remaining ^1^H, as well as ^13^C and ^15^N nuclei, providing slower relaxation rates and sharper line widths [55]. Protonated methyl groups often serve as key sensitive probes of protein structure and dynamics and are of special value in NMR studies [8]. Such, perduterated [^2^H, ^13^C, ^15^N; ^1^H-Ile δ1, Leu-δ, Val-γ]-enriched protein sample provide not only significant improvement of resolution and reductions of peak linewidths, but also allow analysis of backbone and some sidechain methyl resonance assignments that can be used for structural and dynamic studies of important enzymes like RCL.

Although the structures and dynamics of glycosylated proteins are both important and feasible, as a first step towards such studies it is useful to complete resonance assignments and structural analysis of the non-glycosylated protein. As shown in the [^1^H, ^15^N]-TROSY-HSQC and [^1^H, ^13^C]-HSQC spectra in Fig. 5, glycosylation increased protein rotational correlation times (τ_c_) and conformational heterogeneity, and can cause extensive line-broadening in the NMR spectra. In many cases, the sugar moiety is not essential for structural integrity or functional activity of the protein and can be trimmed [56]. Indeed, most crystallographic and/or NMR studies of glycoproteins have been completed using non-glycosylated versions of these proteins. For example, although *P. pastoris* has been used to prepare protein samples for more than 90 NMR structures deposited in the PDB database, in some of these studies glycosylation was suppressed by mutation of N-linked Asn glycosylation sites to Gln [19, 57, 58], while in other cases glysosylation was not characterized [59]. In the solution NMR structure of recombinant Ber e1, a major allergen from Brazil nut containing four disulfide bonds, the sample produced using *P. pastoris* included three O-linked glycans which were retained for the NMR studies. This heterogenous glycosylation, which is not observed in the native protein purified from Brazil nut, did not prevent the NMR structure analysis, but contributed unnecessary complications and line broadening [60].

In preparing glycoproteins for structural studies, glycans are often removed by glycosidase treatment. This glycosylation is relatively routine for N-linked glycans. Unfortunately, there are no general glycosidases that completely remove all O-linked glycans. In this case a mix of exoglycosidases can be followed by a broad specificity O-glycosidase (Protein Deglycosylation Mix, NEB Inc.; Enzymatic Protein Deglycosylation Kit, Sigma-Aldrich Inc.). However, this process is not very efficient. Following glycosidase treatment, the final products may still contain residual glycan, which complicates NMR spectral data.

Although yields are lower per liter, is also less about 50 % less expensive to produce ^13^C,^15^N-enriched r27RCL using the *E. coli* BL21 *trxB* (DE3) system than when using *P. pastoris*. In our experience, ^2^H,^13^C,^15^N-enriched samples could also be produced in *P. pastoris*, as has been reported by other groups [21, 24, 59]. However, we could not biosynthetically incorporate ^1^H, ^13^C methyl groups into otherwise perdeuterated RCL lipase produced in *P. pastoris*, which is also inefficient as reported by Clark et al. [61].

## Conclusion

In this work we have shown that [^2^H, ^13^C, ^15^N; ^1^H-Ile δ1, Leu-δ, Val-γ]-enriched r27RCL lipase with native disulfide bonds, native tertiary structure, and high specific activity can be produced using the *E. coli* BL21 *trxB* (DE3) system. These samples provide the basis for NMR studies of the structure, dynamics, and function of r27RCL. These results also suggest a potential strategy for NMR studies of secreted, glycosylated proteins like RCL lipase, in which samples are first produced in *E. coli* BL21 *trxB* (DE3) system for NMR resonance assignments and initial structural and dynamic studies. These data then provide the basis for studies of glycosylated protein samples produced in *P. pastoris*.

## Abbreviations

RCL: *Rhizopus chinensis* lipase
mRCL: *Rhizopus chinensis* mature lipase
r27RCL(His)_6_: *Rhizopus chinensis* mature lipase with 6 his-tag at C-terminus and 27 amino acids of the C-terminal of the prosequence
r27RCL: *Rhizopus chinensis* mature lipase with 27 amino acids of the C-terminal of the prosequence
proRCL: *Rhizopus chinensis* lipase with prosequence
MBP-proRCL: *Rhizopus chinensis* lipase with prosequence and MBP-tag at the *N*-terminus
ppRCL: RCL expressed in *P. pastoris*
ecRCL: RCL expressed in *E. coli*
*p*NPP: *p*-nitrophenyl palmitate
BMGY: buffered glycerol complex medium
BMMY: buffered methanol complex medium
YNB: yeast nitrogen base
